# Aberrant Brain Functional Connectivity Strength and Effective Connectivity in Patients with Type 2 Diabetes Mellitus

**DOI:** 10.1155/2021/5171618

**Published:** 2021-11-28

**Authors:** Xi Guo, Su Wang, Yu-Chen Chen, Heng-Le Wei, Gang-Ping Zhou, Yu-Sheng Yu, Xindao Yin, Kun Wang, Hong Zhang

**Affiliations:** ^1^Department of Radiology, The Affiliated Jiangning Hospital of Nanjing Medical University, Nanjing, Jiangsu Province 211100, China; ^2^Department of Endocrinology, The Affiliated Jiangning Hospital of Nanjing Medical University, Nanjing, Jiangsu Province 211100, China; ^3^Department of Radiology, Nanjing First Hospital, Nanjing Medical University, Nanjing, Jiangsu Province 210006, China

## Abstract

Alterations of brain functional connectivity in patients with type 2 diabetes mellitus (T2DM) have been reported by resting-state functional magnetic resonance imaging studies, but the underlying precise neuropathological mechanism remains unclear. This study is aimed at investigating the implicit alterations of functional connections in T2DM by integrating functional connectivity strength (FCS) and Granger causality analysis (GCA) and further exploring their associations with clinical characteristics. Sixty T2DM patients and thirty-three sex-, age-, and education-matched healthy controls (HC) were recruited. Global FCS analysis of resting-state functional magnetic resonance imaging was performed to explore seed regions with significant differences between the two groups; then, GCA was applied to detect directional effective connectivity (EC) between the seeds and other brain regions. Correlations of EC with clinical variables were further explored in T2DM patients. Compared with HC, T2DM patients showed lower FCS in the bilateral fusiform gyrus, right superior frontal gyrus (SFG), and right postcentral gyrus, but higher FCS in the right supplementary motor area (SMA). Moreover, altered directional EC was found between the left fusiform gyrus and bilateral lingual gyrus and right medial frontal gyrus (MFG), as well as between the right SFG and bilateral frontal regions. In addition, triglyceride, insulin, and plasma glucose levels were correlated with the abnormal EC of the left fusiform, while disease duration and cognitive function were associated with the abnormal EC of the right SFG in T2DM patients. These results suggest that T2DM patients show aberrant brain function connectivity strength and effective connectivity which is associated with the diabetes-related metabolic characteristics, disease duration, and cognitive function, providing further insights into the complex neural basis of diabetes.

## 1. Introduction

Type 2 diabetes mellitus (T2DM) is proving to be a major global health burden, affecting over 400 million people worldwide [1]. Insulin resistance and chronic hyperglycemia in synergy with other metabolic aberrations in T2DM can adversely affect various organ systems including the brain, causing a range of microvascular and macrovascular complications which can ultimately lead to the cognitive dysfunction and the increased risk of dementia [2]. Once clinical symptoms of dementia appear, the progression cannot be reversed by current treatments. Therefore, early detection of high-risk factors and biomarkers of brain dysfunction in T2DM patients is crucial for timely prevention and treatment [3, 4]. Actually, subtle abnormities of the brain structure and function have already occurred in the T2DM patients before the clinically measurable cognitive dysfunction [5, 6], but the underlying neural mechanisms have not been fully elucidated.

Neuroimaging using functional MRI (fMRI) provides a noninvasive tool to assess neural functional alterations of vulnerable brain regions, promisingly revealing the neural basis of various diseases. Several studies using rest-state fMRI have reported aberrant spontaneous neural activity [7, 8] as well as functional connectivity (FC) changes of the posterior cingulate cortex [9] and angular gyrus [10] or within a specific brain network [11] in T2DM patients. Based on a graph theory, the whole-brain functional connectivity strength (FCS) is a reliable measurement of network organization to analyze the brain functional connections at the voxel level [12, 13], which has facilitated the investigation of connectivity changes in many diseases such as Alzheimer's disease [14], schizophrenia [15], and T2DM. Previous studies have suggested the alterations in functional connectivity strength and density of multiple brain regions and their correlations with cognitive function in T2DM patients [16–18]. However, these findings were not completely consistent, which may be due to the interference of different complications in patients or differences in sample size and gender composition.

In addition, FCS is known as the degree centrality (DC) of weighted networks, which suggest the vital roles of the brain regions with altered FCS in the information communication of the functional network [19]. Therefore, they can be regarded as seed regions to further study their connections with the other regions of the whole brain. However, most of the previous studies have focused on calculating the FC of these brain regions for further investigation, which may not entirely characterize the information flow in the complex neural network. In fact, the integration of functional network can be studied through both the measurement of FC and effective connectivity (EC). Differently from the FC which measures the connectivity between spatially distant brain regions, EC reveals the flow of information and emphasizes on the directionality and the dynamics of information transmission within functional networks [20, 21], which could provide us a much more comprehensive understanding on the neural mechanism of brain connections. Granger causality analysis (GCA) is a popular method to estimate EC based on multiple linear regression, which investigates whether a past value of one time series could correctly predict the current value of another [22]. Recently, GCA has been applied as an effective tool to reveal the causal effects among brain regions and facilitated neuroimaging studies on various neuropsychiatric diseases [23, 24]. EC based on GCA of the hippocampus in T2DM patients has been explored in a recent study [21], which has revealed the altered EC between the hippocampus and default mode network (DMN) and their relationship with cognitive deficits. However, there was no study to identify the abnormal brain functional connections and directional connectivity in T2DM patients by combining FCS and GCA.

In the present study, to investigate the details of resting-state brain function connectivity alterations and their associations with clinical characteristics in T2DM patients, we firstly used the global FCS analysis to explore seed regions with significant changes in T2DM and then used the GCA to detect directional EC between the seed regions and the other regions in the whole brain. Finally, the relationship between EC and clinical variables including diabetes-correlated metabolic characteristics, disease duration, and cognitive function was further explored in T2DM patients.

## 2. Materials and Methods

### 2.1. Participants

This study was approved by the Ethics Committee of Nanjing Medical University, and written informed consent was acquired from all the participants. A total of 60 patients with T2DM and 33 healthy controls (HC) without diabetes were recruited. The inclusion criteria included age (18-75 years) and right-handedness. All T2DM patients fulfilled the 1999 diagnostic criteria by the World Health Organization [25]. All healthy controls were at a normal general cognitive level, defined by a Montreal Cognitive Assessment (MoCA) score ≥ 26. The exclusion criteria for all subjects included a history of stroke, head trauma, epilepsy or other neuropsychiatric diseases, alcohol or substance abuse, any physical illness (such as tumor), severe hearing or visual impairment, MR imaging contraindications, and poor image quality.

### 2.2. Clinical, Biochemical, and Cognitive Assessments

Clinical information including height, weight, body mass index (BMI), and blood pressure of each participant was collected. For T2DM patients, the onset age and duration of diabetes were also recorded.

Blood samples provided by all participants were collected to measure fasting plasma glucose (FPG), hemoglobin A1c (HbA1c), fasting total cholesterol (TC), triglyceride (TG), high-density lipoprotein (HDL) cholesterol, and low-density lipoprotein (LDL) cholesterol levels. T2DM patients underwent an oral glucose tolerance test (OGTT) and provided the blood samples tested for 1- and 2-hour postprandial plasma glucose (2hPG) and insulin.

The general cognitive level was assessed by the MoCA test, which were performed by a trained neuropsychologist for all participants within 1 month after the MR imaging examinations [26].

### 2.3. Data Acquisition

MR imaging data were acquired using a 3.0-Tesla MR scanner (Magnetom Prisma, Siemens Healthineers, Germany) with a 64-channel head coil. All participants were instructed to relax with their eyes closed, stay awake, and avoid any specific thoughts during the scans. Foam padding which was tight but comfortable was used to minimize head motion, and earplugs were used to attenuate scanner noise. Sagittal 3D T1-weighted images were acquired by Magnetization-Prepared 2 Rapid Gradient-Echo (MP2RAGE) sequence with the following parameters: repetition time (TR) = 5000 ms; echo time (TE) = 2.98 ms; TI1 = 700 ms, flip angle (FA) = 4°, TI2 = 2500 ms, and FA = 5°; field of view (FOV) = 256 mm × 256 mm; slice thickness = 1 mm; and 176 sagittal slices. Resting-state blood-oxygen-level dependent (BOLD) images were acquired using a T2∗-weighted echo planar imaging sequence: TR/TE = 1540/22 ms, FOV = 215 mm × 215 mm, FA = 65°, slice thickness = 2 mm, 60 slices, and 590 volumes.

### 2.4. Grey Matter Volume (GMV) Calculation

Voxel-based morphometry (VBM) toolbox (http://dbm.neuro.uni-jena.de/vbm.html) was used to calculate the GMV of each voxel. Using the standard segmentation model, sagittal 3D T1-weighted images were firstly segmented into the grey matter (GM), white matter (WM), and cerebrospinal fluid (CSF). Then, the GM concentration map was registered to Montreal Neurological Institute (MNI) space by the initial affine registration. Using diffeomorphic anatomical registration through the exponential Lie algebra (DARTEL) technique, GM concentration images were nonlinearly warped and then resampled to a voxel size of 3 × 3 × 3 mm^3^. The relative GMV of each voxel was calculated by multiplying the GM concentration map and the nonlinear determinants derived from the spatial normalization. Finally, the GMV images were then spatially smoothed with an 8 mm × 8 mm × 8 mm FWHM Gaussian kernel.

### 2.5. fMRI Data Preprocessing

BOLD MRI data were preprocessed as the following steps using the Rs-fMRI Data Analysis Toolkit plus (RESTplus, http://restfmri.net/forum/). The first ten time points of the fMRI data of each subject were discarded to ensure the signal to reach equilibrium and the participant to adapt to the scanning noise. Then, realignment was performed to the images for the corrections of head motion between time points. All subjects' BOLD data were within the defined motion thresholds (translational or rotational motion parameters less than 3 mm or 3°). The images were then spatially normalized into the MNI space. After removal of the linear trend of the time courses, band-pass filtering of the functional images was applied with a frequency range of 0.01 to 0.08 Hz. Besides, several nuisance covariates were regressed out including six motion parameters and average BOLD signals of the CSF and WM. Finally, the images were resampled into a 3 × 3 × 3 mm^3^ voxel.

### 2.6. Global Functional Connectivity Strength Analysis

Pearson's correlation coefficients were computed between the BOLD time courses of all pairs of voxels within the global GM mask, generating a global GM functional connectivity matrix for each participant. The computation was conservatively restricted to positive correlations with a threshold above 0.5 to eliminate the weak correlations possibly caused by background noise. The FCS of a given voxel was computed as the sum of functional connections between these voxels with all other voxels in the brain. By dividing the FCS of each voxel by the average FCS of the global voxels, the standardized global FCS maps of each subject were obtained and were then spatially smoothed with an 8 mm × 8 mm × 8 mm FWHM Gaussian kernel. Then, using the RESTplus, a two-sample *t*-test was conducted to compare the differences in global FCS between the two groups with the GMV of each voxel as an additional covariate of no interest, controlling for age, sex, and education (*P* < 0.001, uncorrected).

### 2.7. Granger Causality Analysis

We were able to identify the brain regions with altered FCS values in T2DM patients by global FCS analysis. Furthermore, we hoped to obtain greater insight into the alteration of connections of these brain sites. Therefore, the regions that showed differences in global FSC analysis were extracted as seed regions for further analysis.

In the present study, voxel-wise GCA was implemented to estimate EC by RESTplus software [22]. GCA is a method based on multiple linear regression which describes the Granger casual influence of time series (*X*) on the other time series (*Y*). The regressive models are as follows:

(1) $$\begin{array}{c} Y_{t}=\overset{p}{\underset{k=1}{\sum }}A_{k}X_{\left(t-k\right)}+\overset{p}{\underset{k=1}{\sum }}B_{k}Y_{\left(t-k\right)}+CZ_{t}+E_{t},\\ X_{t}=\overset{p}{\underset{k=1}{\sum }}A_{k}^{\prime }Y_{\left(t-k\right)}+\overset{p}{\underset{k=1}{\sum }}B_{k}^{\prime }X_{\left(t-k\right)}+C^{\prime }Z_{t}+E_{t}^{\prime }. \end{array}$$

In the above model, *X*_*t*_ and *Y*_*t*_ represent two time series, respectively. *A*_*k*_ and *A*_*k*_′ are signed-path coefficients, *B*_*k*_ and *B*_*k*_′ are autoregression coefficients, *E*_*t*_ and *E*_*t*_′ are residual, and *Z*_*t*_ represents covariates. The time series *X*_*t*_ significantly Granger causes the time series *Y*_*t*_ if the signed-path coefficient *A*_*k*_ is significantly larger. Similarly, *Y*_*t*_ can be defined as a significant Granger cause to *X*_*t*_ if the signed-path coefficient *A*_*k*_′ is significantly larger [22].

In the present study, the time series of the seed regions extracted in the FCS analysis were defined as the seed time series *X*, and the time series of all the rest voxels in the brain were time series *Y*. We estimated both the *X* to *Y* and the *Y* to *X* effects. A positive coefficient from *X* to *Y* indicates a positive influence of the activity in region *X* on the activity in region *Y* whereas a negative coefficient from *X* to *Y* indicates that the activity of region *X* exerts an adverse influence on the activity of region *Y*. Then, the generated EC maps were further normalized by Fisher *z*-transformation. Finally, a two-sample *t*-test was conducted to compare the differences in EC of the seed regions between the two groups, controlling for age, sex, and education (*P* < 0.001, uncorrected).

### 2.8. Statistical Analysis

Gender data was examined with a *χ*^2^ test using the Statistical Package for the Social Sciences version 22.0 (SPSS, Chicago, IL, USA). The other demographic and clinical variables were analyzed with two-sample *t*-tests.

To investigate the associations between the altered EC and the clinical variables, diabetic characteristics, and cognitive function, the mean EC values of significant regions were extracted and spearman correlation coefficients were then calculated in T2DM patients using SPSS 22.0 with a threshold of *P* < 0.05.

## 3. Results

### 3.1. Demographics, Clinical, Biochemical, and Cognitive Data

T2DM patients and HC were matched for age, gender, and education. Compared with HC, T2DM patients had significantly higher fasting plasma glucose, HbA1c, systolic blood pressure (SBP), and TG levels. And there were no differences in diastolic blood pressure, TC, HDL, and LDL levels between the two groups. For general cognition status, T2DM patients performed relatively worse on MoCA, but no significant difference was observed between T2DM and HC (Table 1).

### 3.2. Global FCS Changes in T2DM Patients

**Figure 1:**
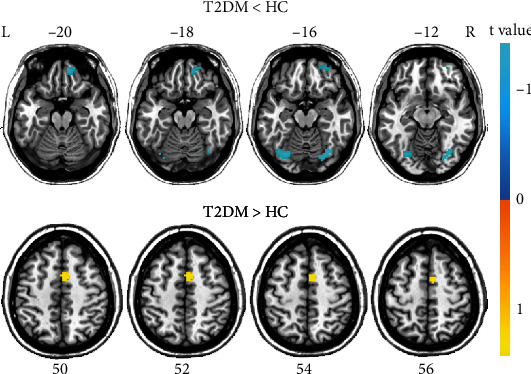
Group differences in global FCS between patients with type 2 diabetes and healthy controls after correction for GMV (*P* < 0.001, uncorrected). HC: healthy controls; L: left; R: right; T2DM: type 2 diabetes mellitus.

Compared with HC, T2DM patients exhibited decreased FCS in the fusiform gyrus bilaterally, the right superior frontal gyrus (SFG, AAL: Frontal_Sup_Orb_R), and right postcentral gyrus, as well as increased FCS in the right supplementary motor area (SMA) (*P* < 0.001, uncorrected) (Figure 1 and Table 2).

### 3.3. EC Changes in T2DM Patients

The brain regions showing differences in global FSC analysis were extracted as seed regions for the further GCA, and the results showed significant EC changes of the left fusiform gyrus and right SFG; there were no significant alterations in the EC of other three seed regions.

**Figure 2:**
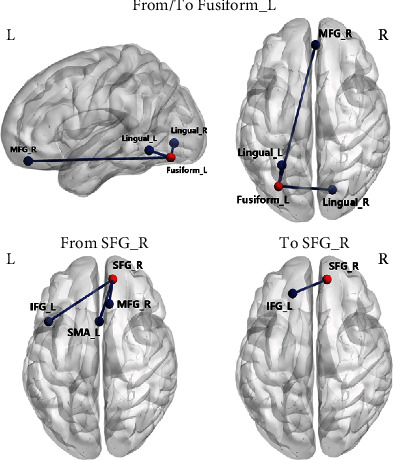
Altered effective connectivity of left fusiform and right superior frontal gyrus in type 2 diabetes compared with healthy control (*P* < 0.001, uncorrected). IFG: inferior frontal gyrus; L: left; MFG: medial frontal gyrus; R: right; SFG: superior frontal gyrus; SMA: supplementary motor area.

In particular, compared with HC, T2DM patients exhibited significantly increased EC from the left fusiform gyrus to right medial frontal gyrus (MFG) and significantly decreased EC from the left fusiform gyrus to the lingual gyrus bilaterally. In addition, T2DM patients compared with HC also showed increased EC from the lingual gyrus bilaterally to the left fusiform gyrus, as well as decreased EC from the right MFG to the left fusiform gyrus (*P* < 0.001, uncorrected) (Figure 2 and Table 3).

Compared with HC, T2DM patients exhibited significantly increased EC from right SFG to the left inferior frontal gyrus (IFG), right MFG, and left SMA and decreased EC from left IFG to right SFG (*P* < 0.001, uncorrected) (Figure 2 and Table 3).

### 3.4. Correlation Analysis Results

**Figure 3:**
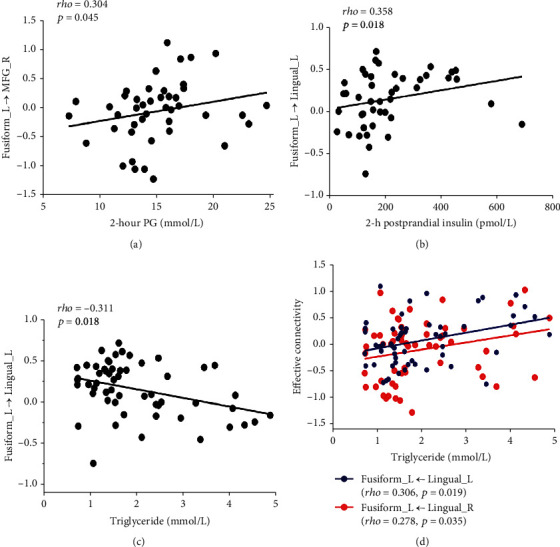
Significant correlations between abnormal effective connectivity of left fusiform and clinical characteristics in type 2 diabetes (*P* < 0.05).

**Figure 4:**
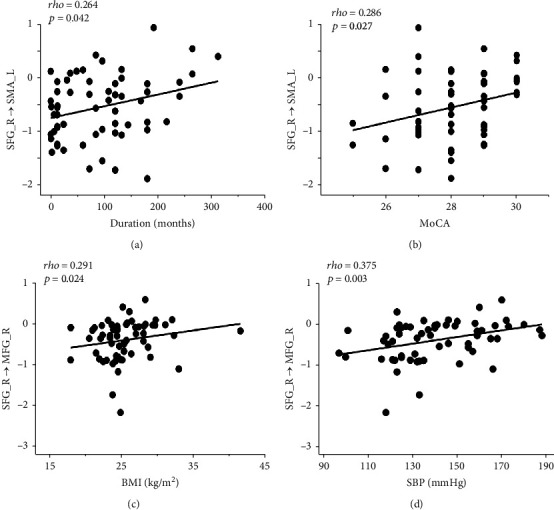
Significant correlations between abnormal effective connectivity of right superior frontal gyrus and clinical characteristics in type 2 diabetes (*P* < 0.05). MFG: medial frontal gyrus; MoCA: Montreal Cognitive Assessment; SBP: systolic blood pressure; SFG: superior frontal gyrus; SMA: supplementary motor area.

The correlations between the significantly changed EC and the clinical variables, diabetic characteristics, and cognitive function are shown in Figures 3 and 4.

In T2DM patients, there were positive correlations between the triglyceride and the abnormal EC from the lingual gyrus bilaterally to the left fusiform gyrus (left: rho = 0.360, *P* = 0.19; right: rho = 0.278, *P* = 0.035) and negative correlation between the triglyceride and the abnormal EC from the left fusiform gyrus to left lingual gyrus (rho = −0.311, *P* = 0.018). 2hPG was positively correlated with the abnormal EC from the left fusiform gyrus to right the MFG (rho = 0.304, *P* = 0.045), and 2-hour insulin was positively correlated with the abnormal EC from the left fusiform gyrus to the left lingual gyrus (rho = 0.358, *P* = 0.018) (Figure 3). In addition, the abnormal EC from the right SFG to the right MFG was positively correlated with BMI (rho = 0.291, *P* = 0.024) and SBP (rho = 0.375, *P* = 0.003). And the abnormal EC from the right SFG to the left SMA was positively correlated with disease duration (rho = 0.264, *P* = 0.042) and MoCA (rho = 0.286, *P* = 0.027) (Figure 4).

## 4. Discussion

To our knowledge, it is the first study to explore brain dysfunctional connectivity in T2DM patients by integrating the FCS and GCA methods. Specifically, T2DM patients showed reduced and increased FCS of the visual cortex, frontal, and sensorimotor regions. In addition, abnormal EC between the fusiform gyrus and the frontal gyrus, the occipital lobe was identified and associated with diabetes-related characteristics (e.g., insulin and plasma glucose levels) in T2DM patients, while the abnormal EC between SFG and several regions including the frontal and motor areas was correlated with disease duration and cognitive function.

In the present study, T2DM patients exhibited decreased FCS in the bilateral fusiform gyrus, decreased EC from the bilateral lingual gyrus to the left fusiform gyrus, and increased EC from the left fusiform gyrus to the bilateral lingual gyrus, as well as increased EC from the right MFG to the left fusiform gyrus and decreased EC from the left fusiform gyrus to the right MFG. The fusiform gyrus is important for the high-level object recognition [27] and is linked with various neural pathways of processing of visual food cues [28]. And the lingual gyrus is anatomically adjacent to fusiform, which is also crucial for the vision information and word processing. The results were consistent with multiple studies [8, 18, 29], suggesting the vision-related region in the occipital lobe is most vulnerable to T2DM. Interestingly, triglyceride was negatively correlated with the decreased outflow from the left fusiform gyrus to the left lingual gyrus and positively correlated with the increase inflow from the left fusiform to the bilateral lingual gyrus. Hypertriglyceridemia is a common symptom of diabetes which was also found in T2DM patients in our study; the correlation results indicate the important roles of the fusiform and lingual gyrus in the neural pathways of lipid metabolism. In addition, a positive correlation was found between the 2 h postprandial insulin and the decreased inflow from the left fusiform gyrus to the left lingual gyrus, which is interpretable as previous research reported that the plasma insulin level is associated with cue-induced appetite at both neural and behavioral levels, which can affect activation of the fusiform gyrus in response to visual processing [30, 31]. Furthermore, 2hPG was positively correlated with the increased inflow from the left fusiform gyrus to the right MFG. This finding implies that the abnormal EC between the two regions may contribute to the impaired glycose tolerance, as the MFG is part of the dorsolateral prefrontal lobe, which plays a key role in diet control, food craving, and metabolic control functions [32, 33]. Taken together, as a chronic metabolic disease, T2DM is closely related to many unhealthy lifestyle habits including obesity caused by excessive eating; the abnormal effective connectivity between the fusiform gyrus and the lingual gyrus and MFG may contribute to the reduced ability to control food intake and abnormal eating habits in T2DM patients, which is manifested as metabolic disturbance such as lipids, glucose, and insulin.

Our study also observed decreased FCS of the right SFG, increased EC from the right SFG to the left IFG, right MFG, and left SMA, and decreased EC from the left IFG to the right SFG. The frontal lobe is the most advanced part of the brain development and is generally considered a vital region mainly responsible for high-order cognitive control [34]. Specifically, SFG is thought to contribute to higher cognitive functions and particularly for working memory [35], which was demonstrated to be anatomically connected with the MFG and IFG through arcuate fibers [34]. Therefore, the results indicate the disrupted internal functional integration of the frontal lobe. This finding is consistent with previous studies using functional connectivity and graph theoretical network [5, 33, 36, 37], suggesting that the frontal lobe, especially the prefrontal cortex, is a vulnerable region in T2DM patients. In our study, MoCA was positively associated with the increased outflow from the right SFG to the left SMA in T2DM patients. T2DM can increase the risk of cognitive impairment, and some of the patients may even develop to dementia. Previous studies reported that T2DM patients performed worse in executive and memory skills and the SFG was associated with cognitive impairment [37, 38]. The general cognitive level reflected by MoCA scores of T2DM patients in this study was still normal, so the increased outflow from the right SFG to the left SMA may play a compensatory role in maintaining the normal cognitive function. And the similar compensatory mechanism of SFG has also been described in previous research using FCD analysis [16]. Moreover, a positive correlation was found between the disease duration and the increased outflow from the right SFG to the left SMA, which also supports the interpretation of the compensatory mechanism of the brain regions. However, the disease duration of TDM patients varies from firstly diagnosed to decades in our study, which may have an influence on the results. As a previous study [39] reported that the compensatory mechanisms often appear in the prediabetes and the early-stage T2DM (duration < 10 years) before the onset of clinically apparent cognitive dysfunction, with the continuous development of the disease, when the functional reorganization fails and functional network begin to disrupt, the cognitive function will be impaired in the later stage of diabetes. Since the sample size of T2DM patients in our study was relatively small, it is difficult for us to further divide the patients to different groups according to the disease duration. Nevertheless, the findings provide a new perspective of the intercommunication disturbance in frontal regions which occurred before the cognitive dysfunction in T2DM patients. Further investigation is necessary to acquire a more comprehensive understanding of the neural mechanism behind the cognitive impairment.

In addition, decreased FCS of the right postcentral and increased FCS of the right SMA were also found in our study. The right postcentral gyrus is in the location of the somatosensory cortex which participates in somatosensory stimulus processing. Previous studies have shown abnormalities in the structure [40] and function [8, 21] of the somatosensory cortex, which may be related to the neuropathy of diabetes causing sensory impairment [41]. SMA is mainly related to the motor functions, and the increased FCS of this area may be interpreted as a compensation for the FCS loss in other brain regions. Here, we notice that there was no significant difference in the EC of these two brain regions between T2DM patients and HC, which may be partly attributed to the different algorithms of FCS and GCA. FCS is the sum connections between the given voxel and the other voxels in the brain which reflect the vital roles of the brain regions in the network, while EC evaluates the information flow between the seed region and other voxels. The two different algorithms focus on different dimensions and properties of the brain functional network. Therefore, brain regions with significant changes in FCS analysis may not show statistically significant EC alterations with other areas.

There were several limitations in this study. Firstly, it was a cross-sectional study with a relatively small sample; therefore, it cannot assess the progression of functional connectivity alterations. Longitudinal studies with a larger sample size to explore the functional connections in the diabetic brain may better characterize the complex neural basis of T2DM patients in the future. Moreover, only the general cognitive function by MoCA was performed in this study; the evaluation of specific cognitive domains including executive function, attention, and memory should be needful to observe the detailed cognitive decline and their correlations with brain functional connectivity alterations. Finally, most of the T2DM patients recruited in this study have been or are being treated with hypoglycemic drugs or insulin, which may contribute to the brain dysfunction and have potential effects on our results. Therefore, a more detailed study taking into account the effect of medication needs to be conducted in the future.

## 5. Conclusions

In conclusion, the present study demonstrates significant alterations of FCS and directional EC in T2DM patients, correlating with the diabetes-related metabolic characteristics, disease duration, and cognitive function. These findings may improve our understandings and provide an implicative neural basis for the T2DM brain abnormality.

## Figures and Tables

**Table 1:** Demographic and clinical characteristics of the participants.

Characteristics	Patients with type 2 diabetes (*n* = 60)	Healthy controls (*n* = 33)	*P* value
Demographic factors			
Sex (female/male)	21/39	15/18	0.322
Age (years)	58.10 ± 9.87	56.06 ± 7.36	0.302
Education (years)	10.88 ± 3.30	11.85 ± 3.07	0.120
BMI (kg/m^2^)	25.58 ± 3.78	24.36 ± 1.82	0.102
Diabetes-related characteristics			
Disease duration (months)^1^	72 (0~312)	—	—
Onset age (years)^1^	50 (26~72)	—	—
HbA1c (%)	8.35 ± 2.06	5.11 ± 0.55	<0.001^∗^
Fasting glucose (mmol/L)	7.5 ± 2.85	4.92 ± 0.58	<0.001^∗^
1 h postprandial glucose (mmol/L)	12.34 ± 2.72(*n* = 43)	—	—
2 h postprandial glucose (mmol/L)	15.06 ± 3.66(*n* = 43)	—	—
Fasting insulin (pmol/L)	44.96 ± 32.67	—	—
1 h postprandial insulin (pmol/L)	212.06 ± 436.27 (*n* = 43)	—	—
2 h postprandial insulin (pmol/L)	211.94 ± 144.90 (*n* = 43)	—	—
Clinical variables			
Arterial blood pressure			
Systolic BP (mmHg)	140.28 ± 21.14	124 ± 8.9	<0.001^∗^
Diastolic BP (mmHg)	82.12 ± 13.44	72.23 ± 7.8	0.035
Total cholesterol (mmol/L)	4.44 ± 0.95	4.48 ± 0.53	0.094
Triglyceride (mmol/L)	1.99 ± 1.07	1.41 ± 0.20	<0.001^∗^
High-density lipoprotein (mmol/L)	1.07 ± 0.271	1.02 ± 0.23	0.557
Low-density lipoprotein (mmol/L)	2.64 ± 0.75	3.31 ± 3.00	0.118
General cognitive assessment			
MoCA	28.08 ± 1.25	28.5 ± 0.94	0.086

Data are presented as mean ± SD or medians and interquartile ranges (25th–75th percentiles)^1^. BMI: body mass index; BP: blood pressure; HbA1c: hemoglobin A1c; MoCA: Montreal Cognitive Assessment; ^∗^*P* < 0.05 was considered significant.

**Table 2:** Brain regions with significant group differences in FCS.

Regions	Brodmann areas	Cluster size (voxels)	Peak *t* values	Coordinates in MNI (*x*, *y*, *z*)
*Diabetes* < *healthy* *controls*				
Fusiform_R	56	34	3.8	33, -69, -15
Fusiform_L	55	40	4.1	-30, -72, -15
Superior_Frontal_Gyrus_R	6	47	4.3	12, 51, -21
Postcentral_R	58	71	4.3	60, -9, 39
*Diabetes* > *healthy* *controls*				
Supp_Motor_Area_R	20	31	-4.3	6, 3, 54

FCS: functional connectivity strength; MNI: Montreal Neurological Institute; L: left; R: right.

**Table 3:** Brain regions with significant group differences in EC.

Effective connectivity	Cluster size (voxels)	Peak *t* values	Coordinates in MNI (*x*, *y*, *z*)
*From Fusiform_L*			
Lingual_R	26	-3.86	18, -75, -3
Lingual_L	108	-5.18	-27, -54, -9
Medial_Frontal_Gyrus_R	27	3.76	3, 48, -18
*To Fusiform_L*			
Lingual_R	23	3.91	18, -75, -3
Lingual_L	51	5.04	-27, -57, -12
Medial_Frontal_Gyrus_R	23	-3.87	3, 51, -18
*From SFG_R*			
Inferior_Frontal_Gyrus_L	46	4.26	-45, 15, 18
Medial_Frontal_Gyrus_R	64	4.23	9, 30, 39
Supp_Motor_Area_L	29	3.74	0, 15, 51
*To SFG_R*			
Inferior_Frontal_Gyrus_L	129	-4.78	-18, 39, 27

MNI: Montreal Neurological Institute; SFG: superior frontal gyrus L: left; R: right.

## Data Availability

The imaging data used to support the findings of this study are included within the article.
